# The human collagen beta(1-O)galactosyltransferase, GLT25D1, is a soluble endoplasmic reticulum localized protein

**DOI:** 10.1186/1471-2121-11-33

**Published:** 2010-05-14

**Authors:** Jolanda MP Liefhebber, Simone Punt, Willy JM Spaan, Hans C van Leeuwen

**Affiliations:** 1 Department of Medical Microbiology, Center of Infectious Diseases, Leiden University Medical Center, 2300 RC Leiden, The Netherlands

## Abstract

**Background:**

Glycosyl transferases transfer glycosyl groups onto their substrate. Localization partially defines their function. Glycosyl transferase 25 domain 1 (GLT25D1) was recently shown to have galactosyltransferase activity towards collagens and another well known substrate, mannose binding lectin (MBL). To gain more insight in the role of galactosylation of lysines in the Gly-X-Lys repeats of collagenous proteins, we investigated the subcellular localization of GLT25D1.

**Results:**

Immunofluorescence analysis of GLT25D1 expressed in the human hepatoma cell line (Huh7), revealed a perinuclear lattice like staining, resembling localization to the endoplasmic reticulum (ER). Possible targeting signals, an N-terminal signal sequence and a C-terminal ER-retention signal, were identified using prediction programs. These signals were then investigated by constructing a series of epitope-tagged forms of GLT25D1 that were analyzed by immunofluorescence and western blotting. In agreement with the predictions our results show that GLT25D1 is directed to the ER lumen as a soluble protein and retained there. Moreover, using two endoglycosidase enzymes EndoH and EndoF, we demonstrate that the putative bi-functional glycosyl transferase itself is a glycoprotein. Additionally we examined co-localization of GLT25D1 with MBL and lysyl hydroxylase 3 (LH3, PLOD3), which is a protein able to catalyze hydroxylation of lysine residues before they can be glycosylated. We demonstrate overlapping localization patterns of GLT25D1, MBL and LH3.

**Conclusions:**

Taken together our data indicate that galactosylation of collagenous proteins by the soluble GLT25D1 occurs in the early secretory pathway.

## Background

Hydroxylation and subsequent glycosylation of lysine residues is a characteristic of collagens and proteins containing a collagen-like region (collectins) [1-3]. These proteins contain repeats of Gly-X-Y motifs, where lysines present at the Y-position can be galactosylated [4,5].

Collagens and collectins are built up of three polypeptide chains, which wind together to form a triple helix. The Gly-X-Y repeats, where X is often a proline, allow tight coiling of the chains as the small glycines fit into sterically restricted spaces where the three chains come together [6].

The function of the lysine linked sugars is not fully understood, but this posttranslational event occurs before triple helix formation [7] and mutations in these residues clearly affect the oligomerization state resulting in aberrant secretion [8]. Mutation of two lysines to arginines in the Gly-X-Lys repeat of mannose binding lectin (MBL), which is involved in neutralization of invading microorganisms by triggering the complement cascade, resulted in inefficient complement activation [8]. At the protein level glycosylated lysines are suggested to play a role in folding, stability and prevention of inter-chain cross-linking [4,8,9].

Lysyl hydroxylase enzymes catalyze the hydroxylation reaction on lysines, after which the residue can by glycosylated [10]. Recently, two new genes responsible for the galactosylation of Gly-X-Y repeats were described. Using affinity chromatography and tandem mass spectrometry, Schegg et al. identified two glycosyltransferase GT25 family members, GLT25D1 and GLT25D2, to encode galactosyltransferases involved in transfer of galactose to hydroxylysine residues in MBL and collagen [11]. Attachment of galactose occurs via the β (1-O) bond to the hydoxylated lysine (Cδ), assigning these proteins to β (1-O) galactosyltransferases.

Most members of glycosyltransferases (GT) are localized to the Golgi, but GTs can also be observed in the cytosol, nucleus, mitochondria, ER, on cellular membranes, secreted from the cell or widely distributed between all these [12]. Their distinct subcellular localization most likely reflects their role in glycosylation pathways. For example, the subsequent arrangement in which glycan synthesis takes place in the Golgi apparatus can often be related to the location of the glycosyltransferases in the cisternae [13]. Here we examined the cellular localization of GLT25D1 relative to one of its reported substrates, MBL, which is produced in liver cells [14]. We also identified ER-targeting signals within GLT25D1 causing it to localize to the early secretory pathway.

## Methods

### Signal predictions

Prediction of signal peptides: http://www.cbs.dtu.dk/services/SignalP/[15]. Prediction of N-glycosylation sites: http://www.cbs.dtu.dk/services/NetNGlyc/.

### Plasmid construction

To construct epitope-tagged GLT25D1 expression plasmids, the sequence was amplified by PCR from GLT25D1 full length clone, IRAKp961P01217Q (imaGenes, Berlin, Germany), using specific primers, see Table 1. The PCR products were digested with *Kpn*I and *Xba*I and ligated into pCDNA3.1mychisB (Invitrogen) similarly digested with *Kpn*I and *Xba*I. This resulted in the construction of expression vectors containing a 10-residue Myc-epitope-tag at its C-terminus. In order to construct C-terminal HA-epitope-tagged GLT25D1 expression constructs (GLT25D1-HA, Δsignal sequence-GLT25D1-HA), the Myc-epitope sequence was *Xba*I - *Pme*I cut and replaced with an *Xba*I - *Pme*I fragment coding for the HA-epitope. For construction of the RDEL at the extreme C-terminus (GLT25D1-MycRDEL, HA-GLT25D1-MycRDEL), the constructs were *Age*I - *Pme*I cut and replaced with a fragment coding for RDEL. For construction of GLT25D1 with an internal Myc-epitope-tag (GLT25D1 FL Myc-int.) after the signal sequence, two annealed oligos with protruding TGCA nucleotides (see table 1) were ligated into the GLT25D1 gene cut with restriction enzyme Sbf-I, which generates complementary ends (CCTGCA|GG).

**Table 1:** Primers used to generate expression constructs

**HA-GLT25D1-Myc (construct 1)**
Forward primer, GTGGGTACCATGTACCCATACGATGTTCCAGATTACGCCGCGGCGGCCCCACGCGC Reverse primer, TAGTCTAGAGAGAGTTCATCCCGGGCAGCAC
**GLT25D1-Myc (construct 2)**
Forward primer, GTGGGTACCATGGCGGCGGCCCCACGCGC Reverse primer as for HA-GLT25D1-Myc
**Δ signal sequence -GLT25D1-Myc (construct 3)**
Forward primer, GTGGGTACCATGGGCGCCGACGCCTACTTC Reverse primer as for HA-GLT25D1-Myc
**HA-GLT25D1 (construct 8)**
Forward primer as for HA-GLT25D1-Myc Reverse primer, TAGTCTAGATCAGAGTTCATCCCGGGCAGCAC
**HA-GLT25D1-ΔRDEL (construct 9)**
Forward primer as for HA-GLT25D1-Myc Reverse primer, TAGTCTAGATCAGGCAGCACTGTCCAGTGG
**GLT25D1-internal tag (construct 10)**
Oligo 1, GGAACAAAAACTCATCTCAGAAGAGGATC*TGCA* Oligo 2, GATCCTCTTCTGAGATGAGTTTTTGTTCC*TGCA*

### Cell culture and transfection

Human hepatoma cell line Huh7 was grown in Dulbecco's Modified Eagle's Medium supplemented with Non-essential amino acids, L-glutamate, Penicillin and Streptavadin. Trypsin was used to subculture the cells. Cells were transfected using Amaxa Cell line Nucleofactor kit T (Lonza), program T016 and 4 μg of DNA.

### Antibodies

The following antibodies were used: anti-PDI (Stressgen), anti-Giantin (Alexis Biochemicals), anti-ERGIC53 (Alexis Biochemicals), anti-LH3 (Abnova), anti-Myc (mouse, immunofluorescence) (Invitrogen), anti-Myc (rabbit, immunofluorescence) (Abcam), anti-Myc (mouse, western blotting) (Roche) and anti-HA (mouse) (Abcam), Donkey-anti-mouse-cy3 (Jackson), Donkey-anti-rabbit-cy3 (Jackson), Goat-anti-mouse-Alexa488 (Invitrogen), Goat-anti-rabbit-Alexa488 (Invitrogen), Goat-anti-mouse-Alexa633 (Invitrogen) and Goat-anti-mouse-HRP (Dako).

### Immunofluorescence

Cells were fixed with 3% paraformaldehyde in PBS (154 mM NaCl, 1.4 mM Phosphate, pH 7.5) 24h post transfection. PFA was quenched using 50 mM NH4Cl in blockbuffer, which contained 5% fetal calf serum (FCS) in PBS. The cells were permeabilized with 0.1% TritonX-100 in blockbuffer and stained with primary antibodies diluted in blockbuffer for 1 h. Next the coverslips were washed with blockbuffer and incubated with secondary antibody diluted in blockbuffer for 1 h. After washing with glycinebuffer (10 mM glycine in PBS), PBS and water, the coverslips were mounted with Prolong mounting medium (Invitrogen). Fluorescence images were captured using Leica TCS SL confocal microscope, 63× Plan Apo oil immersion objective (zoom approximately three times), appropriate filter settings and sequential scan modes. Images were optimized with Adobe Photoshop CS2. Profile plots were generated using ImageJ (NIH).

### SDS-PAGE and western blotting

After separation on SDS-PAGE gels, proteins were transferred to PVDF membranes (Hybond P, GE-Healthcare) using a Semi-Dry blot apparatus (Biorad). Membrane blocking and antibody incubations were performed using 0.5% Tween-20, 5% non-fat, dry milk (Campina) in PBS. Since all secondary antibodies were conjugated to horseradish peroxidase, the proteins were visualized using enzyme-catalyzed chemiluminescence (ECL+, GE-Healthcare) and Typhoon Imager (GE-Healthcare).

### De-glycosylation assay

Cells transfected with GLT25D1-FL-MycHis6 or GLT25D1-ΔSS-MycHis6 were lysed after 16 h in isotonic buffer, which contains 20 mM Tris pH 7, 1 mM MgCl2, 15 mM NaCl, 240 mM sucrose and 10 mM imidazole, using a ball bearing homogenizer (Isobiotec, Heidelberg Germany). Following centrifugation of the cell lysates at 800 g for 10 minutes, supernatants were incubated with cobalt-beads (Talon) for 2 h under continuous rotation. Proteins binding to the beads were eluted using the isotonic buffer now containing 190 mM imidazole. Eluates were split into three portions to de-glycosylate or not. One sample was boiled for 10 min in Laemmli buffer and subsequently treated with Endoglycosidase H (New England Biolabs) in 50 mM sodium citrate for 3, 5 h or overnight at 37°C. Another sample was adjusted to 1% NP40, followed by boiling for 10 min. N-Glycosidase F (EndoF) (Roche) was added to this sample and incubated for 3,5 h or overnight at 37°C. The third sample was directly examined by SDS-PAGE.

### Modular structures of GLT25D1

A multiple sequence alignment of the strongly similar sequences corresponding to GLT25D1 (Swiss-Prot:Q8NBJ5), GLT25D2 (Swiss-Prot:Q8IYK4) and GLT25D3 (Swiss-Prot: Q5T4B2) was created using AlignX (VectorNTI, invitrogen) with the ClustalW algorithm (default settings). This sequence alignment was used as input for the HHpred comparison tool (http://toolkit.tuebingen.mpg.de/hhpred[16]). Selected: database pdb70, Max. PSI-BLAST iterations: 0, other settings default.

The region corresponding to aa 48 - 299 of GLT25D1 produced a hit with the structure of pp-GalNAc-T10 (PDB ID: 2D7I[17], belonging to the CAZY family GT-2/PF00535), probability 92.6, E-value-0.006, P-value 3E-7. The region corresponding to aa 331 - 525 of GLT25D1 produced a hit with the structure of the catalytic Domain Of Mouse Manic Fringe (PDB ID: 2J0A[18], belonging to the CAZY family GT-31 [19], probability 43, E-value-0.15, P-value 0.00076.

The indicated sequences of GLT25D1, including gaps and insertions created by the HHpred, were structurally modeled onto the backbone of these structures, 2D7I and 2J0A, using Protein Homology/analogY Recognition Engine [20].

## Results

### GLT25D1 localizes mainly to the ER

**Figure 1:**
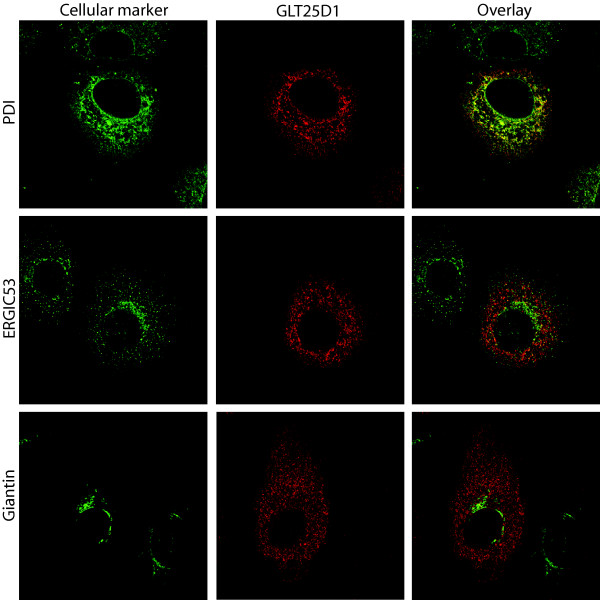
**Subcellular localization of Glycosyltransferase 25 domain 1 protein**. Huh7 cells were transfected with full length Glycosyltransferase 25 domain 1 containing an internal Myc-tag (GLT25D1) (See for more details Methods). After 24 h of expression, cells were fixed and subjected to immunofluorescence analysis. Cells were double labeled for GLT25D1 by anti-Myc (shown in red) and for the cellular markers (shown in green). ER is visualized by anti-protein disulfide isomerase (PDI) (top panels), ER-Golgi intermediate compartment by anti-ERGIC53 (middle panels) or Golgi by anti-Giantin (bottom panels). Yellow observed in the overlay indicates overlap of red and green signal. Pearsons correlation of PDI and GLT25D1 is 0.58, ERGIC53 and GLT25D1 is 0.13, and Giantin and GLT25D1 is 0.04.

To elucidate to which particular compartment (tagged-) GLT25D1 was targeted, we compared the localization with subcellular markers by immunofluorescence in human hepatoma cell line Huh7 (Figure 1). Dual staining of the internally Myc-tagged GLT25D1 transfected cells using antibody to the ER-resident protein, protein disulphide isomerase (PDI) together with the Myc-antibody demonstrated virtually complete overlap (yellow coloring), showing clear ER localization of GLT25D1 (Figure 1). Colocalization can be quantified using Pearsons correlation, where -1 means no overlap and 1 complete colocalization. The Pearsons correlation calculated for PDI compared to GLT25D1 was 0.58, meaning considerable overlapping patterns. We furthermore examined the ER-Golgi intermediate compartment (ERGIC) and Golgi, using ERGIC53 and Giantin respectively. Figure 1, however shows only minor overlap between GLT25D1 and ERGIC/Golgi with Pearsons correlations of 0.13 and 0.04 respectively. Detailed inspection of many transfected cells showed no altered ER or Golgi distribution in GLT25D1 transfected compared to non-transfected cells (Figure 1 and data not shown). Together these data indicate GLT25D1 mainly localizes to the early secretory pathway; particularly ER.

### Predictions of GLT25D1

**Figure 2:**
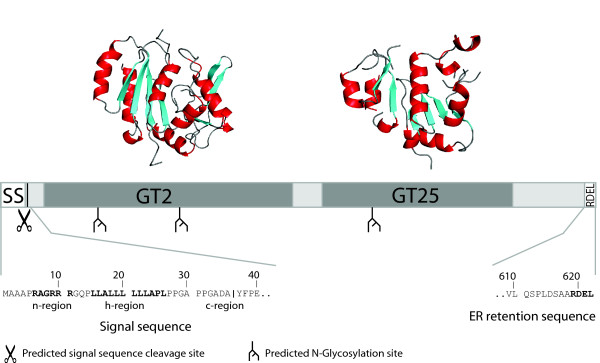
**Schematic representation of Glycosyltransferase 25 domain 1, including various predicted motifs**. Top: Homology based structural models of the tandem glycosyltransferase domains. Bottom: Linear representation of GLT25D1 with possible signal sequence, ER retention signal and N-linked glycosylation sites indicated. Verification and discussion of these predictions are described in this article.

**Figure 3:**
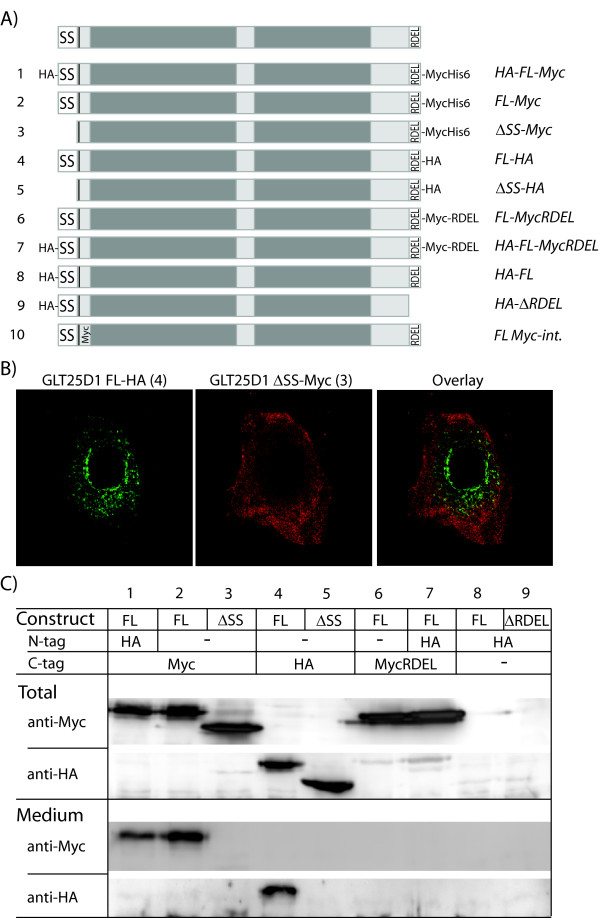
**Characterization of signals in Glycosyl transferase 25 domain 1, suggesting ER residence**. A) Summary of GLT25D1 expression constructs. HA indicates Hemagglutinin epitope tag; Myc refers to Myc epitope tag; FL denotes a full-length protein; RDEL represents carboxy-terminal Arg-Asp-Glu-Leu sequence; SS stands for signal sequence and Δ is followed by deleted part of the protein. B) Co-transfected Huh7 cells with constructs GLT25D1 FL-HA and GLT25D1 ΔSS-Myc were subjected to immunofluorescence analysis. Signal of FL-HA is shown in green and of ΔSS-Myc in red. C) The constructs 1 to 9 shown in A were transfected into Huh7 cells. The total cell lysates (Total) and the culture medium (Medium) from these cells were analyzed by SDS-PAGE and western blot. Using anti-bodies against the N- and C-terminal tags of the constructs, anti-HA and anti-Myc, the proteins were detected.

A first step towards identifying the mechanisms responsible for directing GLT25D1 to the ER is defining subcellular localization signals by using prediction algorithms (See Methods). Consistent with our observed ER localization, we noted that the N-terminal 40 amino-acids encompass a potential ER signal sequence, including a hydrophobic core region (h-region), a positively charged N-terminus (n-region) and a potential cleavage site between position 36 and 37 (Figure 2). Furthermore, upon examining the C-terminus of the protein we noticed that the four extreme C-terminal residues Arg-Asp-Glu-Leu (RDEL) strongly resemble the tetrapeptide sequence KDEL, which causes retention in the ER [21]. Both predictions point to possible insertion of GLT25D1 into the ER and, if cleavage occurs, release from the ER membrane into the ER lumen. In order to investigate these potential targeting signals a series of deletions from the N-terminus as well as the C-terminus, including epitope tags on the opposite side, were made to observe their effect on localization (Figure 3A).

### N-terminal signal sequence targets GLT25D1 to the ER

We first studied the localization of GLT25D1 with the putative signal sequence deleted (GLT25D1-ΔSS-Myc-tagged) compared to full-length GLT25D1 (GLT25D1 FL-HA-tagged). As shown in Figure 3B little overlap was evident with the full-length protein and the ΔSS protein, the latter displaying a diffuse cytosolic staining. This strongly suggests that the extreme N-terminus is a genuine signal sequence and targets the protein to the ER. Furthermore when the N-terminal signal sequence of GLT25D1 was fused to GFP (SS-GFP), a reticular expression pattern was observed in a few cells (data not shown).

### Cleavage of the GLT25D1 signal sequence

Next to the signal sequence a potential cleavage site is predicted (c-region, Figure 2). Cleavage of this N-terminal signal of GLT25D1 was analyzed by blotting total cell lysate of cells transfected with N-terminally HA-tagged GLT25D1 construct (Figure 3A: HA-FL-Myc, construct 1). As shown in Figure 3C, the N-terminal HA-tagged GLT25D1 protein (Figure 3C: lane1, Total, anti-HA) was not visible with an anti-HA antibody, suggesting that the N-terminus was cleaved off. This construct is also tagged at the C-terminus with a Myc-tag that was detected in the total cell lysate, showing that the protein was indeed correctly expressed (Figure 3C: lane1, Total, anti-Myc). When Huh7 cells were transfected with GLT25D1 only Myc-tagged at the C-terminus (Figure 3A: FL-Myc, construct 2) identical protein size was detected compared to GLT25D1 tagged on both sides (Figure 3C: lane1 and 2, Total, anti-Myc). This furthermore indicates that the N-terminal signal sequence must be cleaved, since both proteins have a distinct N-terminus, which should have resulted in a different size in the absence of cleavage. Note the size difference between the full length GLT25D1 construct and the GLT25D1 construct with a deleted signal sequence (Figure 3C: compare lanes 2 and 3, Total, anti-Myc), which is caused by protein glycosylation and will be explained below. Our findings are furthermore confirmed with other constructs, FL-MycRDEL, HA-FL-MycRDEL, HA-FL and HA-ΔRDEL (Figure 3C: lanes 6, 7, 8 and 9, Total, anti-Myc and anti-HA). The N-terminal HA-tag is not detected in lanes 7, 8 and 9 and a similar molecular weight of the constructs HA-FL-MycRDEL and FL-MycRDEL is observed. Hence, apparent by these results, the signal sequence of GLT25D1 is cleaved.

### Carboxy terminal RDEL retains GLT25D1 in the ER

Proteins targeted to the ER that lack a specific retention signal are secreted or transported to the cell-membrane. Since the hydrophobic signal sequence of GLT25D1 is clearly cleaved off and its potential hydrophobic membrane anchor lost, the protein could be secreted in the cell culture medium. In contrast, if the C-terminal residues RDEL represent an ER retrieval signal, GLT25D1 would be retained. We therefore examined the presence of the tagged GLT25D1 constructs in the cell culture medium. The constructs HA-FL and HA-FL with RDEL deleted (HA-FL-ΔRDEL) could not be shown to be present in the medium (Figure 3C: lanes 8 and 9, Medium, anti-HA), because the HA recognition epitope tag is cleaved off together with the signal sequence. Transfected HA-FL-Myc, FL-Myc and FL-HA could be detected with a C-terminal tag in the medium of Huh7 cells (Figure 3C: lanes 1, 2, and 4, Medium, anti-Myc and anti-HA). However addition of an epitope tag (either Myc or HA) at the C-terminus of GLT25D1 could interfere with the RDEL retention function, resulting in (partial) secretion. When we compare localization of GLT25D1-HA tagged and GLT25D1 internally Myc-tagged (GLT25D1 FL Myc-int.), we observe large overlapping patterns (Additional file 1). Assuming that the internal tag reflects in endogenous proteins best, the tagging seems to influence localization only slightly.

In order to preserve the RDEL at the extreme C-terminus, we constructed RDEL at the end of two constructs, HA-FL-MycRDEL and FL-MycRDEL. In contrast to HA-FL-myc and FL-myc, both these constructs could not be detected in the medium (Figure 3C: lanes 6 and 7, Medium, anti-Myc), indicating that the RDEL is an ER-retention signal for GLT25D1. Additionally, as anticipated protein without the signal sequence ΔSS-Myc and ΔSS-HA, which are located in the cytosol, were not observed in the hepatocyte cell culture medium (Figure 3C: lanes 3 and 5, Medium, anti-Myc and anti-HA). Taken together these data clearly indicate that RDEL retrieves GLT25D1 to the ER.

Cleavage and retrieval of GLT25D1 could indicate that the protein is soluble. To confirm this we performed a membrane floatation gradient experiment (Additional file 2). As marker for integral membrane proteins we used Calnexin and PDI was used as a type of ER soluble protein. As anticipated, Calnexin is observed in the membrane floatation fractions with a peak signal present in fraction 7, while PDI is mainly found in the non-floating, bottom fractions. The bulk of GLT25D1 is observed in the bottom fractions of the gradient, indicating the protein is not an integral membrane protein.

### GLT25D1 is N-linked glycosylated

**Figure 4:**
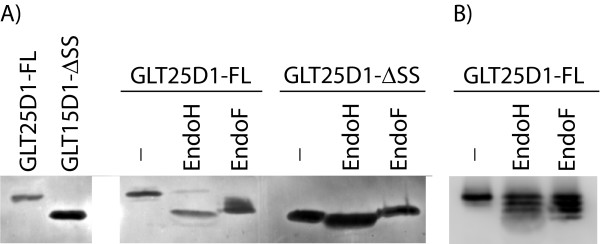
**N-linked glycosylation of Glycosyl transferase 25 domain 1**. A) Full length GLT25D1 (FL) or GLT25D1 with the signal sequence deleted (ΔSS) constructs were transfected into Huh7 cells. Both constructs were carboxy terminal tagged with MycHis6, to purify proteins using Co^2+^-beads and to detect in western blotting. Cell lysates are shown in the first two lanes after separation on SDS-PAGE and western blotting using an antibody against Myc. The purified proteins were subjected to either none (-) or Endoglycosidase H (EndoH) or F (EndoF) treatment (See for more details Methods). The results for FL protein are shown in lanes 3 to 5 and for ΔSS protein in lanes 6 to 8. B) Partial de-glycosylation treatment of GLT25D1 full length (FL).

Unexpectedly there is a substantial size difference between full-length GLT25D1 proteins, which are cleaved in the ER, and proteins with a deleted ER signal sequence (ΔSS) (Figure 4A: compare lane 1 with 2 and Figure 3C: compare lanes 1, 2 and 4 with lanes 3 and 5, Total, anti-Myc and anti-HA). The signal sequence deletion constructs were made starting at the predicted signal cleavage site and should therefore be of equal size. The higher molecular weight of the processed full-length proteins might be explained by posttranslational modifications occurring in the ER, which can not take place on proteins with a deleted signal sequence contained in the cytosol. A potential modification increasing the molecular weight of a protein occurring in the ER is Asn-linked glycosylation. To assess glycosylation, we employed digestion with Endoglycosidase H and F (EndoH, EndoF), which cleave high mannose-containing oligosaccharides. GLT25D1 was purified from Huh7 cells transfected with FL-Myc (Figure 3A: construct 2) or ΔSS-Myc (Figure 3A: construct 3), then treated with EndoH/EndoF, separated by SDS-PAGE and subjected to western blot analysis (Figure 4A) (See for more details Methods). Figure 4A shows that after de-glycosylation the full-length protein decreases in molecular weight similar to the delta-signal sequence protein. This illustrates that the size difference is caused by glycosylation. When the full-length protein was only shortly incubated with EndoH or EndoF, we could discern, besides the full-length protein, three additional products (Figure 4B). These four bands likely correspond to three glycosylations of GLT25D1.

### GLT25D1 colocalizes with mannose binding lectin and lysyl hydroxylase

**Figure 5:**
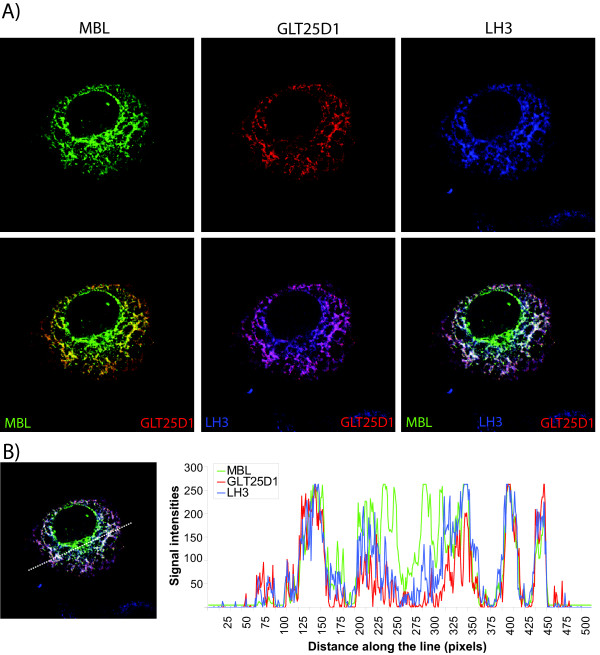
**Co-localization of Glycosyl transferase 25 domain 1 with Mannose binding lectin and Lysyl hydroxylase 3**. A) Huh7 cells were co-transfected with Mannose binding lectin fused to GFP (MBL-GFP) and full length GLT25D1 tagged with Myc internally (GLT25D1), and analyzed by immunofluorescence after 24 h of expression. MBL was detected directly due to GFP and is shown in green. GLT25D1 and lysyl hydroxylase 3 (LH3) were visualized using antibodies against Myc or LH3 and are presented in red and blue, respectively. When red and green signals overlap this is observed as yellow, red and blue as purple and red, blue and green as white. Pearson correlation of GLT25D1 and MBL is 0.53, GLT25D1 and LH3 is 0.42, and LH3 and MBL is 0.45. B) A profile plot with signal intensities from MBL (green), GLT25D1 (red) and LH3 (blue) along the line drawn in the overlay on the left.

The report by Schegg et al., which elegantly demonstrated that GLT25D1 shows a strong galactosyltransferase activity towards mannose binding lectin (MBL), prompted us to compare the intracellular location of GLT25D1 and its substrate MBL [11]. To correlate the expression of GLT25D1 with MBL in liver cells, where MBL is synthesized, we transfected GFP-tagged MBL and internal Myc-tagged GLT25D1 into Huh7 cells and performed immunofluorescence analysis. In Figure 5A GLT25D1 is shown in red and MBL in green (Figure 5A: top, first two panels), they both have a perinuclear and reticular staining. MBL localizes intracellular predominantly to the ER, though also accumulates in foci (Figure 5A: top, first panel) that were previously shown by Nonaka et al. to be ER exit sites [22]. In accordance with ER localization, substantial co-localization of GLT25D1 with MBL is illustrated by yellow coloring in the overlay (Figure 5A: bottom, first panel).

Hydroxylation of lysines, to which galactosyltransferases transfer galactose, is carried out by lysyl hydroxylases (LH). These enzymes act upstream of glycosyltransferases. We therefore triple labeled the cells with LH3, which is one of the three LH isoforms in humans [10], depicted in blue (Figure 5A: top, third panel). The reticular and perinuclear staining of LH3 is similar to GLT25D1, which is also shown in the overlay of Figure 5A in purple (Pearson correlation: 0.42) (Figure 5A: bottom, second panel). In summary, there seem to be partially overlapping patterns of GLT25D1, MBL and LH3 (Figure 5A: bottom, third panel, white staining). To further substantiate co-localization, we generated an intensity graph of the signals from each of these three proteins (Figure 5B). In the plot we observe similar profiles, indicating comparable sub-cellular localization of GLT25D1, MBL and LH3, with exception of structures close to the nucleus containing MBL.

## Discussion

The human protein GLT25D1 was reported to have galactosyltransferase activity towards mannose binding lectin (MBL), transferring galactose to hydroxylysine residues in the Gly-X-Lys repeats [11]. In this report we examined the GLT25D1 localisation by immunofluorescence and made deletion mutants of GLT25D1 to ascertain whether the predicted subcellular targeting signal sequences were functional (Figure 2 and Figure 3A). It should be noted that exogenous over-expression might influence localization due to saturation of the transport machinery. We performed our studies with a human hepatoma cell line, Huh7, because MBL is produced mainly by liver cells [14]. Additionally, we confirmed by mass spectrometry analysis that GLT25D1 is normally expressed in these cells (Observed peptides are shown in Additional file 3). Moreover identical results were obtained in an additional cell line VERO, derived from monkey kidney epithelial cells. We found that GLT25D1, after being targeted to and cleaved in the ER (Figure 1 and Figure 3C), appears primarily in the ER most likely due to a functional ER retention signal, RDEL (Figure 3C). Although GLT25D1 gets secreted when a C-terminal tag is present, no secretion was found when the extreme four amino acids are RDEL, showing that these represent a functional ER retention signal (Figure 3C). This modified but related to KDEL carboxyl-terminal tetrapeptide has been shown to direct intracellular retention for several other proteins [21]. Moreover GLT25D1 is highly sensitive to Endoglycosidase H, which is able to cleave non-complex N-linked oligosaccharides present in the ER [23]. This not only shows targeting to the ER lumen, but also ER-retention of the soluble GLT25D1 (Figure 4A).

We furthermore demonstrate by partial digestion with Endoglycosidase H or F that at least three asparagine residues become N-glycosylated (Figure 4B). In accordance with the number of glycosylated forms we distinguish, the NetNglyc server exactly predicted three asparagines to be N-glycosylated (residues 96, 184, 404) (Figure 2), suggesting that all these three residues are modified.

GLT25D1 is able to galactosylate MBL and other collagens [11]. Interestingly, we observe colocalization between GLT25D1 and MBL. Intracellularly MBL forms oligomers before it moves from the ER to the Golgi apparatus [8] and is secreted to serve as an activator of the lectin complement pathway. Binding of MBL to carbohydrates on pathogens not only can mediate an innate immune response towards microbes, but more and more data indicate a potential defense against viruses [24,25]. The envelope glycoproteins of human immunodeficiency virus (HIV), Ebola and Influenza A virus were demonstrated to attach to serum MBL [26-28]. Additionally a subcellular interaction between HIV glycoprotein gp120 and MBL was shown [22]. The presence of GLT25D1 mainly in the ER, where it colocalizes with MBL and LH3, suggests that galactosylation by GLT25D1 occurs early in bio-synthesis before being transported to the Golgi. This would be in line with the results of Heise et al., which demonstrate that glycosylation of MBL continues while transport to the Golgi complex is blocked by Brefeldin A treatment [8].

Before galactosylation of the lysine in the Gly-X-Lys repeat, the lysine is hydoxylated. LH3 has hydroxylase, galactosyltransferase and glucosyltransferase activity (Reviewed in [10]). When performing domain prediction analysis of GLT25D1 (see Methods), we noticed that GLT25D1 is a modular glycosyltransferase (GT) composed of two GT family members, with the C-terminal one related to glycosyltransferase of family 25 (hence its name) and an additional N-terminal domain which displays distant relation to family GT2 transferases (Figure 2 and Additional file 4). In both domains the conserved DxD motif, which binds one of the ribose hydroxyl groups, could be observed (See Additional file 4; Bernard Henrissat personal communications). Other examples of modular GTs are Heparin synthase, Chondroitin synthase and hyaluronan synthase, each involved in addition of alternating sugars, and each containing an N-terminal GT2 domain [29]. Part of this GT2 domain of GLT25D1 surprisingly shows sequence homology to LH3, which suggests that they might function similarly either in substrate recognition or in glucosyltransferase activity.

It is interesting to note that in a search for tumor-specific markers both GLT25D1 and LH3 genes were upregulated in a large majority of human malignancies [30], but the biological pathway involved is unknown. The location of a particular glycosyltransferase in the cell clearly defines its function and biological importance. Most glycosyltransferases reside in the Golgi, where glycan synthesis takes place in a sequential order [12,13]. The presence of GLT25D1 early in the secretory pathway indicates its enzyme activity is displayed there, confirming that collagens and collectins are likely to be glycosylated in the ER before they move to the Golgi apparatus and are secreted. Yet the exact role of glycosylated hydroxylysine residues in collagenous proteins is still poorly understood. Nonetheless, our results strengthen the hypothesis that glycosylation of collagens and collectins occurs early in their biosynthesis.

## Conclusions

Our experiments show that GLT25D1 is a soluble protein present in the lumen of the ER and is cleaved after its signal sequence and is N-glycosylated at three positions. The occurrence of GLT25D1 early in the secretory pathway, mainly ER, suggests that collagens and collectins are likely to be galactosylated before trafficking to the Golgi and are secreted.

## Authors' contributions

JMPL performed all biochemical experiments, participated in the design of the study and wrote the manuscript. SP constructed mutants and critically read the manuscript. WJMS was involved in revising the manuscript critically and participated in supervision of the study. HCL drafted the manuscript, supervised and designed the study. All authors read and approved the final manuscript.

## Supplementary Material

Additional file 1 **Localization of GLT25D1-HA compared to internally Myc-tagged GLT25D1**. Huh7 cells were co-transfected with full length GLT25D1-HA (GLT25D1 FL-HA) and internally Myc-tagged full length GLT25D1 (GLT25D1 FL Myc-int.). 24 h after transfection the cells were subjected to immunofluorescence analysis. GLT25D1 FL-HA and GLT25D1 FL Myc-int. were detected by antibodies against HA (green) and Myc (red), respectively. Pearson correlation of GLT25D1 FL-HA and GLT25D1 FL Myc-int. is 0.83. Click here for file

Additional file 2 **GLT25D1 is a luminal ER protein**. After 24 h, Huh7 cells transfected with GLT25D1 FL-HA were subjected to sucrose density gradient centrifugation. Cell lysates were loaded under a sucrose gradient from 10-80% w/v. Fractions were taken from top (fraction 1) to bottom (fraction 12) and separated by SDS-PAGE, followed by immunoblot analysis for Calnexin and PDI (protein disulfide isomerase). GLT25D1 FL-HA was visualized using an antibody against HA-epitope. Click here for file

Additional file 3 **GLT25D1 peptide coverage**. Huh7 cell lysates were subjected to two-dimensional polyacrylamide gel electrophoresis, followed by silverstaining. Spots, corresponding to Mw 72 kDa and isoelectric point ~7 were digested with trypsin and analysed by ESI tandem MS. The obtained peptide sequences from GLT25D1 are shown in bold in the primary sequence of the protein. Click here for file

Additional file 4 **Sequence alignment of human GLT25D1, LH3(PLOD3) and close homologues**. Alignment was generated using the ClustalX program. DXD motif is indicated (red box). Yellow indicates putative glycosyltransferase folds. FeII_Oxy indicates a predicted Fe(II)-dependent oxygenase superfamily. Numbers indicate amino acid position. Click here for file
