# Supplementary figures and images for: The human collagen beta(1-O)galactosyltransferase, GLT25D1, is a soluble endoplasmic reticulum localized protein
Source: BMC Cell Biol. 2010 May 14;11:33. doi: 10.1186/1471-2121-11-33 (PMC2877668; doi:10.1186/1471-2121-11-33)

Additional file 1

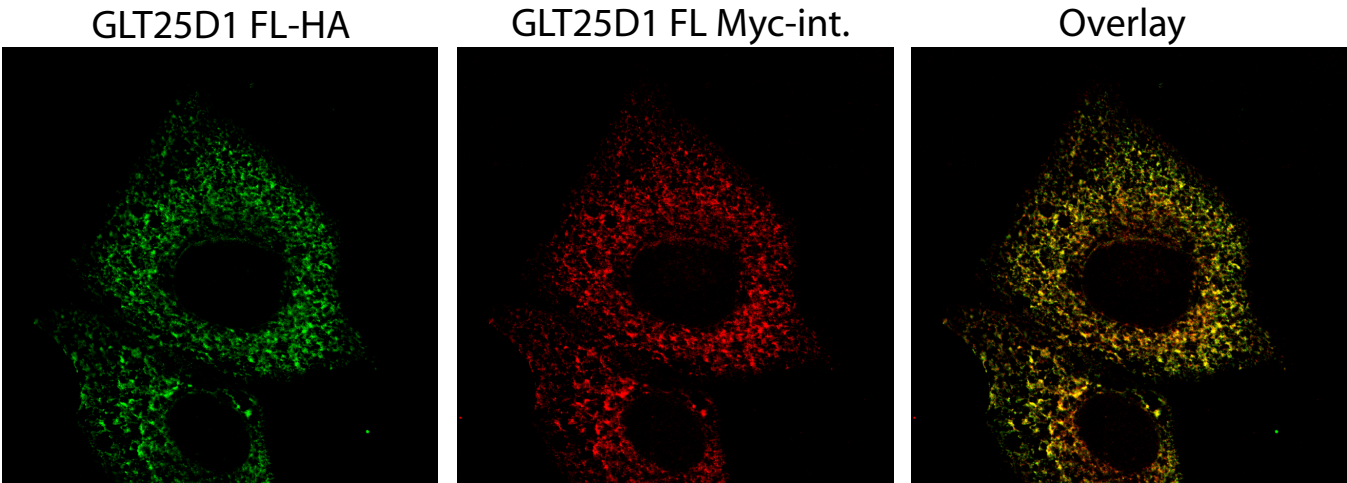

Supplement: Additional file 1 — Localization of GLT25D1-HA compared to internally Myc-tagged GLT25D1. Huh7 cells were co-transfected with full length GLT25D1-HA (GLT25D1 FL-HA) and internally Myc-tagged full length GLT25D1 (GLT25D1 FL Myc-int.). 24 h after transfection the cells were subjected to immunofluorescence analysis. GLT25D1 FL-HA and GLT25D1 FL Myc-int. were detected by antibodies against HA (green) and Myc (red), respectively. Pearson correlation of GLT25D1 FL-HA and GLT25D1 FL Myc-int. is 0.83. [file 1471-2121-11-33-S1.PDF]

Additional file 2

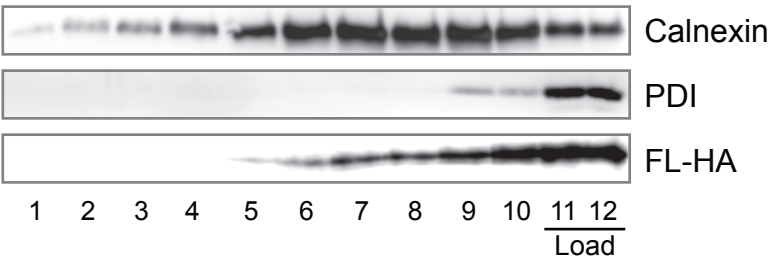

Supplement: Additional file 2 — GLT25D1 is a luminal ER protein. After 24 h, Huh7 cells transfected with GLT25D1 FL-HA were subjected to sucrose density gradient centrifugation. Cell lysates were loaded under a sucrose gradient from 10-80% w/v. Fractions were taken from top (fraction 1) to bottom (fraction 12) and separated by SDS-PAGE, followed by immunoblot analysis for Calnexin and PDI (protein disulfide isomerase). GLT25D1 FL-HA was visualized using an antibody against HA-epitope. [file 1471-2121-11-33-S2.PDF]

Additional file 4

LH3

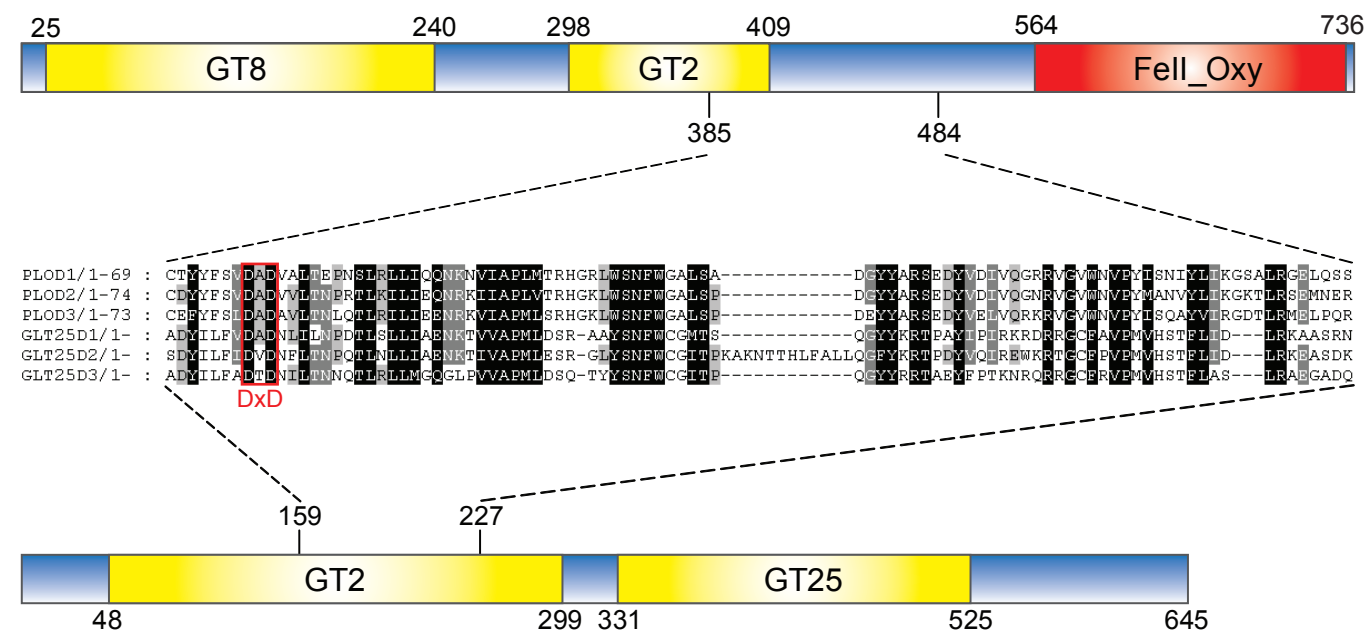

GLT25D1

Supplement: Additional file 4 — Sequence alignment of human GLT25D1, LH3(PLOD3) and close homologues. Alignment was generated using the ClustalX program. DXD motif is indicated (red box). Yellow indicates putative glycosyltransferase folds. FeII_Oxy indicates a predicted Fe(II)-dependent oxygenase superfamily. Numbers indicate amino acid position. [file 1471-2121-11-33-S4.PDF]
