# Supplementary material for: The human collagen beta(1-O)galactosyltransferase, GLT25D1, is a soluble endoplasmic reticulum localized protein
Source: BMC Cell Biol. 2010 May 14;11:33. doi: 10.1186/1471-2121-11-33 (PMC2877668; doi:10.1186/1471-2121-11-33)
Supplement: Additional file 3 — GLT25D1 peptide coverage. Huh7 cell lysates were subjected to two-dimensional polyacrylamide gel electrophoresis, followed by silverstaining. Spots, corresponding to Mw 72 kDa and isoelectric point ~7 were digested with trypsin and analysed by ESI tandem MS. The obtained peptide sequences from GLT25D1 are shown in bold in the primary sequence of the protein. [file 1471-2121-11-33-S3.PDF]

Additional file 3

MAAAPRAGRRRGQPLLALLLLLLAPLPPGAPPGADAYFPEER**WSPESPLQAPRVLIALLA**
RNAAHALPTTLGALERLRHPRERTALWVATDHNMDNTSTVLREWLVAVK**SLYHSVEWRPA**
EEPRSYPDEEGPKHWSDSRYEHVMKLRQAALKSARDMWADYILFVDADNLILNPDTLSLL
IAENK**TVVAPMLDSR**AAYSNFWCGMTSQGYYKR**TPAYIPIR**KRDRRGCFAVPMVHSTFLI
DLRKAASRNLAFYPPHPDYTWSFDDIIVFAFSCKQAEVQMYVCNK**EEYGFLPVPLR**AHST
LQDEAESFMHVQLEVMVK**HPPAEPSRFISAPTK**TPDKMGFDEVFMINLRRRQDRRERMLR
ALQAQEIECRLVEAVDGKAMNTSQVEALGIQMLPGYRDPYHGRPLTKGELGCFLSHYNIW
KEVVDRGLQKSLVFEDDLR**FEIFFK**RR**LMNLMR**DVER**EGLDWDLIYVGRKRMQVEHPEK**A
VPRVRNLVEADYSYWTLAYVISLQGAR**KLLAAEPLSK**MLPVDEFLPVMFDKHPVSEYK**AH**
FSLRNLHAFSVEPLLIYPTHYTGDDGYVSDTETSVVWNNEHVKTDWDRAKSQK**MREQQAL**
SREAKNSDVLQSPLDSAARDEL
